# Association of Hospitalised Infection With Socioeconomic Status in Patients With Rheumatoid Arthritis Receiving Biologics or Tofacitinib: A Population-Based Cohort Study

**DOI:** 10.3389/fmed.2021.696167

**Published:** 2021-07-12

**Authors:** Hsin-Hua Chen, Ching-Heng Lin, Chen-Yu Wang, Wen-Cheng Chao

**Affiliations:** ^1^Department of Medical Research, Taichung Veterans General Hospital, Taichung, Taiwan; ^2^Division of Allergy, Immunology and Rheumatology, Department of Internal Medicine, Taichung Veterans General Hospital, Taichung, Taiwan; ^3^Institute of Biomedical Science and Rong Hsing Research Centre for Translational Medicine, Chung Hsing University, Taichung, Taiwan; ^4^Department of Industrial Engineering and Enterprise Information, Tunghai University, Taichung, Taiwan; ^5^School of Medicine, China Medical University, Taichung, Taiwan; ^6^Institute of Public Health and Community Medicine Research Center, National Yang-Ming University, Taipei, Taiwan; ^7^Big Data Center, Chung Hsing University, Taichung, Taiwan; ^8^Department of Healthcare Management, National Taipei University of Nursing and Health Sciences, Taipei, Taiwan; ^9^Department of Public Health, College of Medicine, Fu Jen Catholic University, New Taipei City, Taiwan; ^10^Department of Critical Care Medicine, Taichung Veterans General Hospital, Taichung, Taiwan; ^11^Department of Nursing, Hung Kuang University, Taichung, Taiwan; ^12^Department of Computer Science, Tunghai University, Taichung, Taiwan; ^13^Department of Automatic Control Engineering, Feng Chia University, Taichung, Taiwan

**Keywords:** rheumatoid arthritis, biologics, infection, risk factors, socioeconomic status

## Abstract

**Objectives:** Use of biologics or targeted synthetic disease-modifying anti-rheumatic drugs (b/tsDMARDs) is associated with infection in patients with rheumatoid arthritis (RA). Socioeconomic status is substantial in infectious diseases; however, the impact of socioeconomic status on risk for infection in patients with RA receiving b/tsDMARD remains unclear.

**Methods:** We used the 2003–2017 Taiwanese National Health Insurance Research Database to identify patients with RA receiving b/tsDMARDs. A Cox regression analysis was used to estimate the associations of covariates with the risk of hospitalised infection shown as hazard ratios (HRs) with 95% confidence interval (CIs).

**Results:** We identified 7,647 RA patients who started their first bDMARD/tsDMARD treatment. Log-rank analyses demonstrated the association between age (*p* < 0.001), urbanisation (*p* = 0.001), the insured amount (*p* = 0.021), and the hospitalisation. Cox proportional regression analyses showed that age was independently associated with hospitalised infection in a dose–response manner, whereas a high-income category had an inverse association (HR 0.48, 95% CI 0.23–0.96). Hospitalisation for infection within 5 years was a strong risk factor (HR 5.63, 95% CI 1.91–16.62), and living in a rural area tended to be a risk factor (HR 1.76, 95% CI 0.98–3.14) for incident hospitalised infection.

**Conclusions:** This study showed the crucial impacts of age, socioeconomic status, and history of infection on hospitalised infection in patients with RA receiving b/tsDMARDs. These findings highlight the largely ignored role of socioeconomic status in risk stratification among patients receiving b/tsDMARDs for RA.

## Background

Rheumatoid arthritis (RA) affects 0.5–1% of the global population and ranks as the 42nd highest contributor to the global disability with an enormous economic impact due to devastating arthritis and disability (1–3). A remarkable advance in the management for RA has been achieved after the development of biological disease-modifying anti-rheumatic drugs (bDMARDs), so-called biologics, and targeted synthetic DMARD (tsDMARD) in the past two decades (4, 5). The efficacy of b/tsDMARDs in the management of RA is well-established; however, an increased risk for infection remains a major concern (6–11). Accumulating evidence has shown the essential impact of socioeconomic status on the risk for infection in general populations (12, 13), but few studies have addressed the impact of socioeconomic status on risk for infection in patients with RA receiving b/tsDMARDs (10). In the present population-based study in Taiwan, we enrolled biologics-naïve patients with RA and received b/tsDMARDs between 2003 and 2017 to address risk factors, including income and urbanisation level, for infection requiring hospitalisation.

## Methods

### Ethics Approval

The Institutional Review Board (IRB) of Taichung Veterans General Hospital (IRB TCVGH number: CE19038A) approved this study. Informed consent was waived, given that all of the personal information had been anonymised before data analyses.

### Study Design

The study is a retrospective cohort study.

### Data Source

This study utilised the 2003–2017 claim data from the National Health Insurance Research Database (NHIRD). Taiwan implemented an obligatory National Health Insurance (NHI) program in 1995. The NHI program has since then covered over 99.6% of the population in Taiwan in 2017 (14). The data of the NHIRD contains comprehensive claims data regarding the information on registration, demographic characteristics, residence, prescriptions, diagnosis, examinations, procedures, medical expenditures, outpatient services, and inpatient services. The Bureau of NHI (BNHI) has improved the accuracy of claims data in the NHIRD by checking original medical records regularly (15). The BNHI established a registry for catastrophic illness patients (RCIP) for those who have severe or major diseases, such as RA and cancers, and patients with a certificate for the RCIP were exempt from copayment. Notably, a certificate for RCIP was only issued after validation by at least two qualified specialists through a comprehensive review of original medical charts. We utilised multiple NHIRD datasets to conduct this study, including 2003–2017 outpatient and inpatient claims files and RCIP enrolment files.

### Study Subjects

We identified patients with biologics-naïve patients with RA newly treated with b/tsDMARD during 2003–2017 as study subjects. We defined mutually exclusive categories based on the initiated treatment: (1) tumour necrosis factor inhibitors (TNFis) (etanercept, adalimumab, and golimumab), (2) non-TNFi bDMARDs (abatacept and tocilizumab), and (3) tsDMARD (tofacitinib). The index date was the first date of bDMARD/tsDMARD prescription.

### Outcome

The outcome was the time from the index date to the time of the first inpatient visit with a diagnosis of infection (Supplementary Table 1). We defined the censored date as 90 days after the last date of bDMARD/tsDMARD prescription, switching to another b/tsDMARD, December 31, 2017 (the last date of the data used in this study), or the time of withdrawal from the NHI for any reason, such as leaving or death, whichever came first. The follow-up duration was from the index date to the date of hospitalised infection occurrence or from the index date to the censored date. We calculated the incidence of hospitalised infection by dividing the number of incident hospitalised infection cases by the sum of the follow-up durations.

### Covariates

Potential predictors for the risk of hospitalised infection included baseline socioeconomic status, sex, age at the index date, comorbidities, medication use within 6 months before the index date, and medication use during the follow-up period. The studied medications included non-steroidal anti-inflammatory drugs (NSAIDs), glucocorticosteroids, methotrexate, sulfasalazine, leflunomide, hydroxychloroquine, and immunosuppressants (i.e., cyclosporine, azathioprine). The presence of comorbidities was defined as having ≥3 outpatient visits or ≥1 hospitalisation with the corresponding International Classification of Diseases-9-Clinical Modification (ICD-9-CM) code or ICD-10-CM code within 1 year before the index date. We used the urbanisation level and insured amount to explore the impact of socioeconomic status. The urbanisation level of the patients' residence was categorised into two levels based on population density (people/km^2^), population ratio of elderly subjects aged ≥65 years, population ratio of subjects with educational levels of college or above, population ratio of agricultural workers, and number of physicians/100,000 subjects (16). The payroll-related insured amount was categorised into an ordinal variable with three levels in accordance with the payroll bracket categories of NHI program in Taiwan.

### Statistical Analysis

We presented continuous variables as a mean ± standard deviation and categorical variables as a percentage of patients. We examined the differences in continuous variables by Student's *t*-test and categorical variables by Pearson's χ^2^ test. The primary dependent variable in this study was hospitalised infection incidence, and the incidence rates (per 100,000 person-year) and incidence rate ratios (IRRs) were analysed. The Kaplan–Meier method was used to compare the cumulative incidence of hospitalised infection among patients categorised by age, urbanisation status, and insured amount. We quantified the associations between covariates and the risk of hospitalised infection by estimating hazard ratios (HRs) with 95% confidence intervals (CIs) using Cox proportional regression analysis after adjusting for potential confounders. We considered a two-tailed *p* < 0.05 as statistically significant. We performed all statistical analyses by SAS statistical software, version 9.3 (SAS Institute, Inc., Cary, NC, USA).

## Results

### Characteristics of the Study Population

A total of 7,647 patients with RA and who received their first b/tsDMARDs between 2003 and 2017 were eligible for analyses. Of them, 6,063 patients received TNFi treatment (etanercept *n* = 2,862; adalimumab *n* = 2,289; golimumab *n* = 912), 1,065 received non-TNFi bDMARDs (tocilizumab *n* = 502; abatacept *n* = 563), and 519 patients were treated with tsDMARD (tofacitinib).

The mean age was 53.9 ± 12.8 years; 76.3% of them were female. The duration between diagnosis of RA and initiation of b/tsDMARDs was 2.9 ± 2.8 years, while the duration from initiation of b/tsDMARDs to hospitalised infection was 2.9 ± 2.6 years. A distinct socioeconomic status was found among patients with RA and received b/tsDMARDs, with 75.1% of them living in an urban area, and 41.4% of them having had a relatively high income. Notably, ~1% of the enrolled patients had hospitalised infection within 5 years. Table 1 further summarises comorbidities, medications prior to the initiation of b/tsDMARDs, and concomitant medications with the use of b/tsDMARDs (Table 1).

**Table 1:** Characteristics of enrolled subjects with RA receiving b/tsDMARDs.

	**All**	**Etanercept**	**Adalimumab**	**Golimumab**	**Tocilizumab**	**Abatacept**	**Tofacitinib**
	**7,647**	**2,862**	**2,289**	**912**	**502**	**563**	**519**
**Demographic data**							
Age at initiating b/tsDMARDs, years	53.9 ± 12.8	53.3 ± 12.7	53.6 ± 12.8	54.0 ± 12.6	54.6 ± 12.1	56.7 ± 13.2	55.3 ± 12.7
Gender, female	5,836 (76.3)	2,182 (76.2)	1,729 (75.5)	708 (77.6)	387 (77.1)	424 (75.3)	406 (78.2)
Disease duration before b/tsDMARDs	2.9 ± 2.8	2.5 ± 2.4	2.8 ± 2.6	3.4 ± 3.3	3.1 ± 3.0	3.5 ± 3.2	3.8 ± 3.6
Follow-up duration after b/tsDMARDs	2.9 ± 2.6	3.9 ± 2.9	3.0 ± 2.5	1.9 ± 1.5	1.6 ± 1.2	2.0 ± 1.4	1.1 ± 0.8
**Urbanisation status**							
Urban	5,741 (75.1)	2,088 (73.0)	1,710 (74.7)	698 (76.5)	394 (78.5)	445 (79.0)	406 (78.2)
Rural	1,906 (24.9)	774 (27.0)	579 (25.3)	214 (23.5)	108 (21.5)	118 (21.0)	113 (21.8)
**Insured amount, New Taiwan dollars**							
<19,200	1,978 (25.9)	800 (28.0)	584 (25.5)	205 (22.5)	106 (21.1)	149 (26.5)	134 (25.8)
19,200–22,800	2,525 (33.0)	952 (33.3)	767 (33.5)	296 (32.5)	157 (31.3)	193 (34.3)	160 (30.8)
>22,800	3,144 (41.1)	1,110 (38.8)	938 (41.0)	411 (45.1)	239 (14.6)	221(39.3)	225 (43.4)
**Hospitalised infection within 5 years**	75 (1.0)	36 (1.3)	20 (0.9)	7 (0.8)	3 (0.6)	5 (0.9)	4 (0.8)
**Comorbidities**							
Hypertension	1,819 (23.8)	679 (23.7)	517 (22.6)	235 (25.8)	108 (21.5)	150 (26.6)	130 (25.1)
Diabetes mellitus	736 (9.6)	260 (9.1)	213 (9.3)	87 (9.5)	61 (12.2)	63 (11.2)	52 (10.0)
Pulmonary disease	539 (7.1)	203 (7.1)	152 (6.6)	50 (5.5)	41 (8.2)	63 (11.2)	30 (5.8)
Chronic kidney disease	158 (2.1)	51 (1.8)	28 (1.2)	21 (2.3)	18 (3.6)	26 (4.6)	14 (2.7)
Chronic liver disease	405 (5.3)	162 (5.7)	119 (5.2)	52 (5.7)	20 (4.0)	31 (5.5)	21 (4.1)
Viral hepatitis	339 (4.4)	143 (5.0)	96 (4.2)	26 (2.9)	22 (4.4)	35 (6.2)	17 (3.3)
**Medications before b/tsDMARDs**							
Methotrexate (cumulative dose/week, 2.5 mg)	4.1 ± 2.0	4.2 ± 2.0	4.1 ± 2.1	4.0 ± 1.9	3.8 ± 2.1	3.8 ± 2.0	4.1 ± 1.9
Sulfasalazine (cumulative dose/day, 500 mg)	1.8 ± 1.6	2.0 ± 1.6	1.9 ± 1.6	1.7 ± 1.6	1.5 ± 1.6	1.6 ± 1.6	1.6 ± 1.6
Leflunomide (cumulative dose/day, 50 mg)	0.07 ± 0.13	0.06 ± 0.12	0.09 ± 0.14	0.07 ± 0.12	0.06 ± 0.12	0.07 ± 0.13	0.05 ± 0.11
Hydroxychloroquine (cumulative dose/day, 200 mg)	1.2 ± 0.8	1.2 ± 0.8	1.1 ± 0.8	1.1 ± 0.8	1.2 ± 0.8	1.2 ± 0.7	1.2 ± 0.7
Cyclosporin/azathioprin (cumulative DDD/day)	0.04 ± 0.13	0.05 ± 0.13	0.05 ± 0.14	0.03 ± 0.12	0.04 ± 0.12	0.04 ± 0.13	0.02 ± 0.09
Prednisolone equivalent (mg/day)	6.2 ± 6.5	6.8 ± 7.2	6.2 ± 6.3	5.8 ± 5.7	6.0 ± 7.3	5.9 ± 5.1	4.5 ± 4.0
**Concomitant medications with b/tsDMARDs**							
Methotrexate (mg/week)	3.8 ± 4.5	3.5 ± 2.2	3.7 ± 3.6	4.13 ± 4.99	3.7 ± 6.7	3.52 ± 2.73	5.3 ± 10.6
Sulfasalazine (mg/day)	119.1 ± 2,492.8	1.1 ± 1.5	150.6 ± 2,787.3	124.0 ± 2,621.0	335.6 ± 4,320.4	150.3 ± 2,636.8	378.6 ± 4,419.5
Leflunomide (mg/day)	0.4 ± 13.4	0.04 ± 0.1	0.3 ± 8.6	0.7 ± 18.5	1.4 ± 22.5	0.1 ± 0.2	2.2 ± 34.7
Hydroxychloroquine (mg/day)	33.4 ± 590.4	0.8 ± 0.8	32.6 ± 584.5	37.7 ± 641.6	140.3 ± 1198.2	30.9 ± 527.3	108.8 ± 1095.0
Cyclosporin/azathioprin (mg/day)	0.03 ± 0.43	0.02 ± 0.08	0.05 ± 0.68	0.02 ± 0.12	0.02 ± 0.10	0.02 ± 0.11	0.04 ± 0.74
Prednisolone equivalent (mg/day)	5.1 ± 16.1	4.1 ± 5.1	5.2 ± 14.3	5.1 ± 14.3	7.8 ± 37.0	4.5 ± 5.3	7.4 ± 32.0

*Data are presented as mean ± standard deviation and N (%).RA, rheumatoid arthritis; b/tsDMARDs: biologics and targeted synthetic disease-modifying antirheumatic drugs; DDD, defined daily dose*.

### Incidence Rate and IRRs of Hospitalised Infection

Table 2 shows the incidence rates of hospitalised infection and the IRRs with 95% CIs. We found that age affected the incidence of hospitalised infection, and the incidence rates (per 100,000 person-years) were 829 (≥65 group), 285 (46–65 group), and 53 (18–45 group), respectively. The IRR was 15.68 (4.79–51.39) in those older than 65 years and 5.40 (1.66–17.51) in patients whose ages ranged from 45 and 65 years compared with the incidence rate among patients whose ages are between 18 and 45 years. Socioeconomic status also had a crucial impact on incident hospitalised infection. The IRR was 2.50 (1.56–4.01) in patients living in a rural area compared to those living in an urban area, whereas the IRRs were 0.41 (0.18–0.94) in patients with the highest income category compared with patients with the lowest income category. With regard to the effect of distinct b/tsDMARDs on incident hospitalised infection, we found no significant difference in incident hospitalised infection among the distinct b/tsDMARDs (Table 2).

**Table 2:** Incidence rate of infection requiring hospitalisation in patients receiving b/tsDMARDs.

**Variable**	**Total**	**Event (%)**	**Total person-years**	**Incidence rate (/10^5^ years)**	**IRR (95% CI)**	**Log-ran**
						***P***
**Age at initiating b/tsDMARDs, years**						<0.001
18–45	1,735	3 (0.17)	5,677	53	1	
45–65	4,358	37 (0.85)	12,969	285	5.40 (1.66–17.51)	
>65	1,554	30 (1.93)	3,620	829	15.68 (4.79–51.39)	
**Gender**						0.138
Female	5,836	49 (0.84)	17,221	285	1	
Male	1,811	21 (1.16)	5,046	416	1.46 (0.88–2.44)	
**Urban**						<0.001
Urban	5,741	38 (0.66)	16,662	228	1	
Rural	1,906	32 (1.68)	5,605	571	2.50 (1.56–4.01)	
**Insured amount, New Taiwan dollars**		0.017				
<19,200	1,978	25 (1.26)	5,979	418	1	
19,200–22,800	2,525	29 (1.15)	7,024	413	0.99 (0.58–1.69)	
>22,800	3,144	16 (0.51)	9,264	173	0.41 (0.18–0.94)	
**b/tsDMARDs**						0.598
Abatacept	563	3 (0.53)	1,151	261	1	
Etanercept	2,862	34 (1.19)	11,156	305	1.17 (0.36–3.81)	
Adalimumab	2,289	26 (1.14)	6,875	378	1.45 (0.44–4.79)	
Golimumab	912	4 (0.44)	1,747	229	0.88 (0.20–3.92)	
Tocilizumab	502	3 (0.60)	787	381	1.46 (0.30–7.25)	
Tofacitinib	519	0 (0.00)	551	0	NA	

*RA, rheumatoid arthritis; b/tsDMARDs: biologics and targeted synthetic disease-modifying antirheumatic drugs*.

### Risk Factors for Hospitalised Infection Among RA Patients Receiving b/tsDMARDs

**Figure 1:**
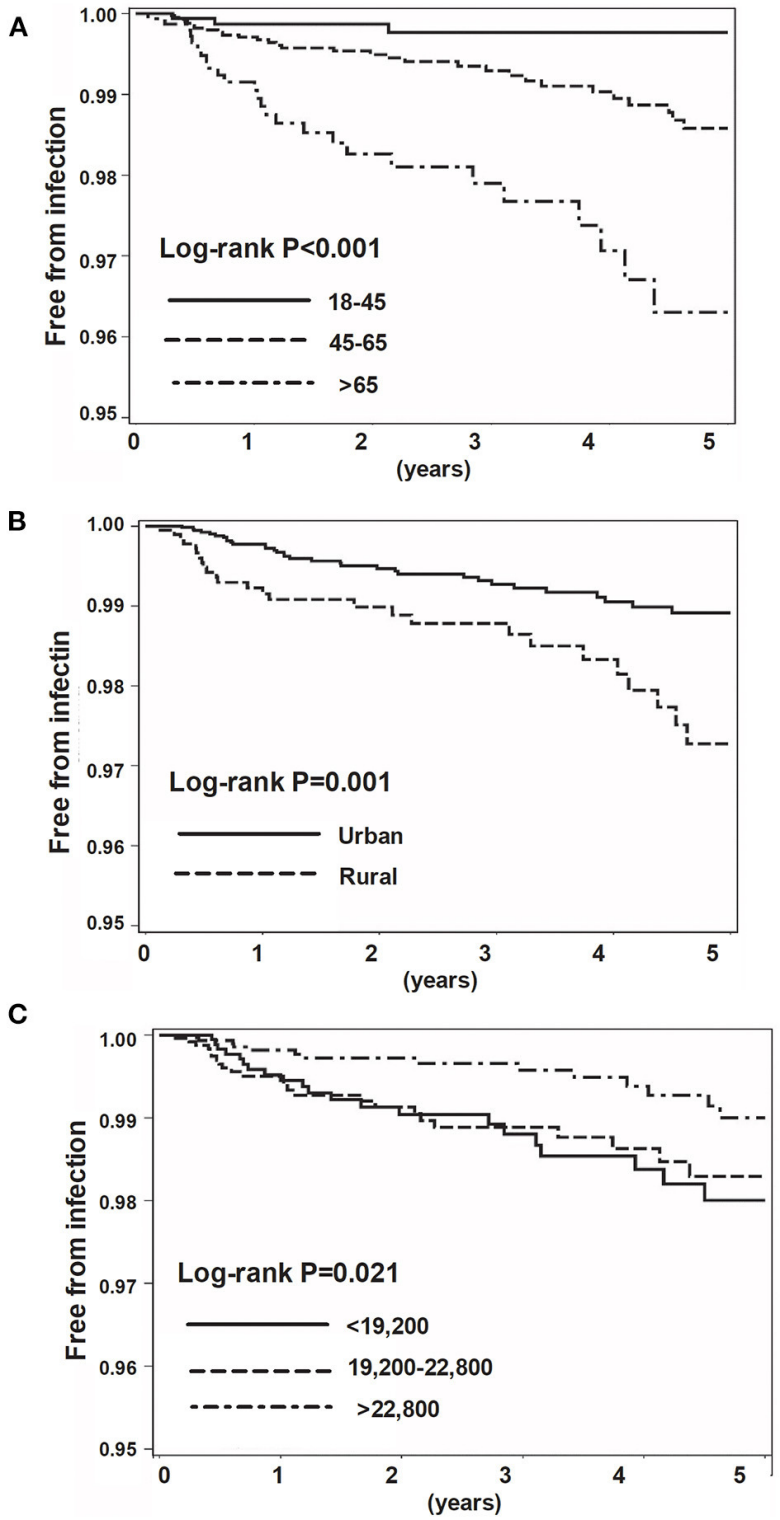
Kaplan–Meier survival curve for incidental hospitalised infection. Categorised by age **(A)**, urbanisation status **(B)**, and insured amount **(C)**.

We then used the log-rank analyses to demonstrate the impact of age, urbanisation status, and income on the incident hospitalised infection (Figure 1). We next estimated the risk for hospitalised infection using univariate and multivariable Cox proportional regression analyses (Table 3). We found that age independently affected the risk for hospitalised infection at a dose–response manner (≥65 age group, HR 6.44, 95% CI 1.82–22.80; 45–64 age group, HR 3.49, 95% CI 1.04–11.70). A high income was inversely associated with the incident hospitalised infection (HR 0.48, 95% CI 0.23–0.96). Living in a rural area tended to be a risk factor for hospitalised infection, although not reaching a statistical significance (HR 1.76, 95% CI 0.98–3.14). We also found that hospitalisations within 5 years (HR 5.63, 95% CI 1.91–16.62) were a robust predictor for incident hospitalised infection after the initiation of b/tsDMARDs in patients with RA. With regard to the effects of previously used and concomitant medication on the hospitalised infection, we noted that the usage of sulfasalazine was somehow inversely associated with hospitalised infection (HR 0.71, 95% CI 0.56–0.91), and the concomitant corticosteroid (HR 1.07, 95% CI 1.05–1.09 prednisolone equivalent per 1 mg/day increment), as well as leflunomide [HR 18.57, 95% CI 1.52–227.06, cumulative dose (50 mg) per day increment], was associated with the incident hospitalised infection. We also analysed data in patients receiving csDMARDs alone. Consistently, the data of 21,361 subjects with RA receiving csDMARDs alone showed that incident hospitalised infection was associated with hospitalisation for infection within 5 years (aHR 2.43, 95% CI 1.35–4.36), whereas a high-income category had an inverse association (aHR 0.43, 95% CI 0.31–0.60) (Supplementary Tables 2–4).

**Table 3:** Crude and adjusted hazard ratios for the association between incident hospitalised infection and variables.

	**HR (95% CI)**	***p value***	**aHR (95% CI)**	***p value***
**Age at initiating b/tsDMARDs, years**				
18–45	Ref.		Ref.	
45–65	4.24 (1.29–13.88)	0.017	3.49 (1.04–11.70)	0.043
>65	12.33 (3.73–40.77)	<0.001	6.44 (1.82–22.80)	0.004
**Gender-male**	1.25 (0.70–2.21)	0.454	0.96 (0.52–1.75)	0.887
**Urbanisation status**				
Urban	Ref.		Ref.	
Rural	2.35 (1.41–3.93)	0.001	1.76 (0.98–3.14)	0.058
**Insured amount, New Taiwan dollars**				
<19,200	Ref.		Ref.	
19,200–22,800	0.95 (0.53–1.71)	0.864	0.68 (0.36–1.27)	0.226
>22,800	0.43 (0.22–0.84)	0.013	0.48 (0.23–0.96)	0.039
**Hospitalised infection within 5 years**	8.61 (3.12–23.77)	<0.001	5.63 (1.91–16.62)	0.002
**Comorbidities**				
Hypertension	2.57 (1.53–4.31)	0.000	1.39 (0.78–2.48)	0.259
Diabetes mellitus	3.10 (1.70–5.65)	0.000	1.83 (0.97–3.45)	0.062
Pulmonary disease	2.69 (1.32–5.47)	0.006	1.31 (0.61–2.83)	0.493
Chronic kidney disease	4.31 (1.56–11.90)	0.005	1.91 (0.63–5.77)	0.251
Chronic liver disease	1.81 (0.72–4.52)	0.205	1.08 (0.39–2.98)	0.887
Viral hepatitis	3.85 (1.82–8.10)	0.000	2.90 (1.20–6.99)	0.018
**b/tsDMARDs**				
Abatacept	Ref.		Ref.	
Etanercept	1.12 (0.34–3.73)	0.854	1.52 (0.45–5.14)	0.500
Adalimumab	1.59 (0.48–5.31)	0.448	2.15 (0.63–7.26)	0.220
Golimumab	0.88 (0.20–3.94)	0.868	1.24 (0.27–5.58)	0.784
Tocilizumab	1.41 (0.29–7.00)	0.673	1.90 (0.38–9.61)	0.439
Tofacitinib	0.00 (0.00–.)	0.979	0.00 (0.00–.)	0.983
**Medications before b/tsDMARDs**				
Prednisolone equivalent, mg/day	1.01 (0.99–1.05)	0.337	0.99 (0.95–1.02)	0.499
Methotrexate (cumulative dose/week, 2.5 mg)	0.76 (0.68–0.85)	<0.001	0.84 (0.70–1.01)	0.057
Sulfasalazine (cumulative dose/day, 500 mg)	0.84 (0.71–0.98)	0.031	0.71 (0.56–0.91)	0.007
Leflunomide (cumulative dose/day, 50 mg)	17.04 (3.43–84.72)	0.001	1.33 (0.11–16.48)	0.825
Hydroxychloroquine (cumulative dose/day, 200 mg)	1.00 (0.73–1.39)	0.979	1.06 (0.69–1.63)	0.782
Cyclosporin/azathioprin (cumulative DDD/day)	3.38 (0.76–15.10)	0.110	1.21 (0.14–10.14)	0.863
**Concomitant medications with b/tsDMARDs**				
Prednisolone equivalent, mg/day	1.06 (1.05–1.08)	<0.001	1.07 (1.05–1.09)	<0.001
Methotrexate (cumulative dose/week, 2.5 mg)	0.84 (0.74–0.95)	0.007	1.11 (0.94–1.32)	0.221
Sulfasalazine (cumulative dose/day, 500 mg)	1.08 (0.90–1.30)	0.394	1.29 (0.99–1.67)	0.059
Leflunomide (cumulative dose/day, 50 mg)	70.18 (13.72–358.98)	<0.001	18.57 (1.52–227.06)	0.022
Hydroxychloroquine (cumulative dose/day, 200 mg)	1.21 (0.88–1.67)	0.239	0.99 (0.66–1.47)	0.950
Cyclosporin/azathioprin (cumulative DDD/day)	9.96 (1.92–51.61)	0.006	2.75 (0.21–36.87)	0.446

*RA, rheumatoid arthritis; b/tsDMARDs, biologics and targeted synthetic disease-modifying antirheumatic drugs*.

## Discussion

To the best of our knowledge, this is the first population-based study to explore the impact of socioeconomic status on the risk for hospitalised infection among patients with RA receiving b/tsDMARDs. We found that age was associated with hospitalised infection in a dose–response manner and prior hospitalised infection was a strong predictor for hospitalised infection after the initiation of b/tsDMARDs in patients with RA. Notably, we found that high socioeconomic status, particularly a high income, was inversely associated with incident hospitalised infection. Additionally, we noted that concomitant use of corticosteroid was also associated with the development of hospitalised infection among patients with RA receiving b/tsDMARDs.

Surprisingly, socioeconomic status has been a proven determinant for incident sepsis in the general population (12, 17); however, the role of socioeconomic status in incident infection among patients with RA receiving b/tsDMARDs is largely unknown although a number of studies have shown that socioeconomic status has a crucial impact on the initiation of b/tsDMARDs (18, 19). Molina et al. investigating 1,209 patients with RA in San Antonio, found that a lower socioeconomic status, defined by education level, household income, and occupation, was associated with a delay of first cDMARDs (8.5 ± 10.2 vs. 6.1 ± 7.9 years; *p* = 0.002) (18). One Swedish study, conducted by Frisell et al. addressed factors associated with the first bDMARD among 9,310 patients with RA and found that patients receiving non-TNFi as the first bDMARD had lower socioeconomic status and were more likely to have recent serious infections than those receiving TNFi (19). The finding of Frisell et al. implicates the potential concern of the development of infection in administering TNFi as the first bDMARD in RA patients with low socioeconomic status. Our recently published study, investigating risk for sepsis in patients receiving TNFi for immune-mediated inflammatory diseases, including RA, ankylosing spondylitis, psoriasis, psoriatic arthritis, Crohn's disease, and ulcerative colitis, identified that risk for sepsis was associated with lower levels of urbanisation and payroll-related insured amount (10). In the present study, we further demonstrated the robust association between hospitalised infection and insured amount in RA patients receiving distinct bDMARDs or tsDMARDs, and we think these findings indicate that socioeconomic status must be considered in infection risk stratification in patients with RA receiving b/tsDMARDs.

Age plays a substantial role in sepsis; however, the impact of age on incident infection in patients with RA appears to vary with studies due to the distinct target to treat strategy, use of b/tsDMARDs, and concomitant GCs (20–22). Increasing evidence has shown that the concern regarding the safety issue of b/tsDMARDs in elderly patients with RA might somehow deprive those patients of optimal disease control and quality of life (20). Tatangelo et al. conducting a population-based study in Canada to explore factors for the duration from the first csDMARDs to the first bDMARDs in patients with RA, reported a positive association between increasing age and longer time to receipt of a bDMARD (21). Furthermore, Black et al. investigated the use of GCs among patients with RA in the United Kingdom primary care research database and found that nearly half of patients with incident RA received GCs and GCs were more frequently prescribed as elderly patients with RA (22). Given that the efficacy of b/tsDMARDs appears unrelated to age, the increased use of GC, instead of b/tsDMARDs, in elderly patients with RA may possibly lead to an increased risk for infection (23). In line with our data, Widdifield et al. conducted a nested case–control study using administrative data across 1992–2010 with 86,039 patients with RA in Ontario, demonstrating that age was an independent predictor for infection requiring hospitalisation or emergency department visit (aOR, 1.05; 95% CI 1.04–1.06) (24). In the present study, we found that the association between age (>65 years) and incident hospitalised infection appears to be higher in RA patients receiving b/tsDMARD (aHR 6.44, 95% CI 1.82–22.80) than those receiving csDMARDs (aHR 3.18, 95% CI 2.19–4.60). This evidence including our data suggests a crucial need for vigilance of infection in elderly patients receiving b/tsDMARDs.

A number of studies have shown a high proportion of patients who were readmitted after discharge from the hospital for the infectious disease (10, 25, 26). Prescott et al. using Medicare claims with 2,617 severe sepsis survivors in the US, found that up to 42.7% severe sepsis survivors were readmitted within 3 months, mainly due to sepsis and pneumonia (25). One Taiwanese population-based study also reported that septic patients had a higher risk of further sepsis than those in age- and sex-matched non-septic controls (35.0 vs. 4.3%) (26). Our previous study found that recent sepsis within 3 months before TNFi initiation was a robust predictor for incident sepsis in patients receiving TNFi in patients with IMIDs (10). In the present study, we further found a robust impact of a history of hospitalised infection on the development of hospitalised infection after receiving b/tsDMARDs among patients with RA. As shown in the aforementioned Swedish study, the physician tends to prescribe non-TNFi, instead of TNFi, as the first bDMARD in RA patients with recent serious infection, indicating the concern of physicians with regard to further infection in prescribing TNFi (19). Therefore, we think that there is a need for vigilance in the follow-up of RA patients who had a history of serious infection and have received b/tsDMARDs; however, more studies are warranted for the optimal strategy in selecting b/tsDMARDs in RA patients with a history of infection, particularly those with a low socioeconomic status.

Intriguingly, we found that the concomitant use of leflunomide was highly associated with the incident hospitalised infection. Given that leflunomide is indicated as the second-line csDMARD in Taiwan after the ineffective/intolerable first-line csDMARD therapy, including methotrexate, sulfasalazine, or cyclosporine, we thus postulate that the high disease activity may potentially underlie the strong association between use of leflunomide and hospitalised infection.

The strength of this study is the use of a nationwide population-based cohort, containing comprehensive data regarding socioeconomic status, to mitigate selection bias. However, some limitations remain to be acknowledged. First is the concern for the accuracy of the diagnosis in claims data; however, the diagnosis of RA was validated by at least two qualified rheumatologists after checking the original medical data. Second, the potential socioeconomic status-associated confounding factors, including the use of tobacco and alcohol, were unavailable in the NHIRD. However, the majority of enrolled patients with RA in the present study were females (76.3%), and the prevalence of tobacco use among females in Taiwan was marked low (2.6%) (27). Third, the study results may not be applied to non-Taiwanese populations with distinct overall socioeconomic status. Fourth, a number of biologics, including anakinra, infliximab, certolizumab, and rituximab (proven as the second-line bDMARD in Taiwan), were not studied given that these biologics were not approved as first-line biologics for RA during the study period in Taiwan. Another limitation is the lack of controls, including disease severity-matched patients with RA but who did not undergo b/tsDMARD therapy and patients receiving b/tsDMARDs for diseases other than RA. However, we have shown consistent findings among patients with RA receiving csDMARDs alone (Supplementary Tables 2–4).

## Conclusion

This nationwide, population-based, cohort study addressed risk factors for hospitalised infection in patients with RA receiving b/tsDMARDs. In line with other studies including our previous study, we found that age and prior hospitalised infection were the robust predictors for hospitalised infection after the initiation of b/tsDMARDs in patients with RA. Notably, we found that high socioeconomic status, including a high income and living in an urbanised area, appeared to be inversely associated with incident hospitalised infection. Moreover, concomitant use of corticosteroid also associated with an increased risk for hospitalised infection. These findings, particularly the identification of the impact of socioeconomic status, should be crucial for risk stratification in patients with RA receiving b/tsDMARDs.

## Data Availability Statement

The original contributions presented in the study are included in the article/Supplementary Material, further inquiries can be directed to the corresponding authors.

## Ethics Statement

The studies involving human participants were reviewed and approved by IRB of Taichung Veterans General Hospital (IRB TCVGH number: CE19038A). The ethics committee waived the requirement of written informed consent for participation.

## Author Contributions

H-HC, C-YW, and W-CC: conceived, designed the experiments, and wrote the paper. H-HC and C-HL: acquired the data. C-HL and W-CC: contributed the materials and analysis tools. All authors contributed to the article and approved the submitted version.

## Conflict of Interest

The authors declare that the research was conducted in the absence of any commercial or financial relationships that could be construed as a potential conflict of interest.
