# Supplementary material for: Association of Hospitalised Infection With Socioeconomic Status in Patients With Rheumatoid Arthritis Receiving Biologics or Tofacitinib: A Population-Based Cohort Study
Source: Front Med (Lausanne). 2021 Jul 12;8:696167. doi: 10.3389/fmed.2021.696167 (PMC8311461; doi:10.3389/fmed.2021.696167)
Supplement: Supplementary file 2 [file Table_2.DOCX]

| **Supplemental table 2. Characteristics of enrolled 21,361 subjects with RA receiving csDMARDs** | |
| --- | --- |
| **Demographic data** |  |
| Age at initiating b/tsDMARDs, years | 54.9±14.3 |
| Gender, female | 16,312 (76.4) |
| Follow-up duration after b/tsDMARDs | 5.0±3.2 |
| **Urbanisation status** |  |
| Urban | 16,201 (75.8) |
| Rural | 5,160 (24.2) |
| **Insured amount, New Taiwan dollars** |  |
| <19,200 | 9,938 (46.5) |
| 19,200-22,800 | 4,840 (22.7) |
| >22,800 | 6,583 (30.8) |
| **Hospitalized infection within 5 years** | 187 (0.9) |
| **Comorbidities** |  |
| Hypertension | 4,869 (22.8) |
| Diabetes mellitus | 2,071 (9.7) |
| Pulmonary disease | 1,567 (7.3) |
| Chronic kidney disease | 450 (2.1) |
| Chronic liver disease | 936 (4.4) |
| Viral hepatitis | 648 (3.0) |
| **Concomitant csDMARDs** |  |
| Methotrexate (mg/week) | 1.5±2.5 |
| Sulfasalazine (mg/day) | 0.8±2.0 |
| Leflunomide (mg/day) | 0.02±0.07 |
| Hydroxychloroquine (mg/day) | 0.7±0.9 |
| Cyclosporin/Azathioprin (mg/day) | 0.01±0.05 |
| Prednisolone equivalent (mg/day) | 3.1±6.8 |
| Abbreviations: RA, rheumatoid arthritis; csDMARDs: conventional synthetic disease-modifying antirheumatic drugs | |
