# Supplementary material for: Association of Hospitalised Infection With Socioeconomic Status in Patients With Rheumatoid Arthritis Receiving Biologics or Tofacitinib: A Population-Based Cohort Study
Source: Front Med (Lausanne). 2021 Jul 12;8:696167. doi: 10.3389/fmed.2021.696167 (PMC8311461; doi:10.3389/fmed.2021.696167)
Supplement: Supplementary file 3 [file Table_3.DOCX]

| **Supplemental table 3. Incidence rate of infection requiring hospitalisation in patients receiving csDMARDs** | | | | | | | |
| --- | --- | --- | --- | --- | --- | --- | --- |
| Variable | Total | Event (%) | Total person-years | Incidence Rate (/10^5^ years) | IRR (95%CI) | Log-rank  P value |  |
| **Age at initiating csDMARDs, years** | | | | |  | <0.001 |  |
| 18-45 | 5,395 | 50 (0.93) | 28,803 | 174 | 1 |  |  |
| 45-65 | 10,833 | 227 (2.10) | 54,810 | 414 | 2.39 (1.76-3.24) |  |  |
| >65 | 5,133 | 255 (4.97) | 22,220 | 1,148 | 6.61 (4.88-8.95) |  |  |
| **Gender** |  |  |  |  |  | <0.001 |  |
| Female | 16,312 | 353 (2.16) | 82,511 | 428 | 1 |  |  |
| Male | 5,049 | 179 (3.55) | 23,322 | 768 | 1.79 (1.50-2.15) |  |  |
| **Urban** |  |  |  |  |  | 0.001 |  |
| Urban | 16,201 | 369 (2.28) | 80,201 | 460 | 1 |  |  |
| Rural | 5,160 | 163 (3.16) | 25,631 | 636 | 1.38 (1.15-1.66) |  |  |
| **Insured amount, New Taiwan dollars** | | | | |  | <0.001 |  |
| <19,200 | 9,938 | 306 (3.08) | 47,916 | 639 | 1 |  |  |
| 19,200-22,800 | 4,840 | 150 (3.10) | 23,705 | 633 | 0.99 (0.82-1.20) |  |  |
| >22,800 | 6,583 | 76 (1.15) | 34,212 | 222 | 0.35 (0.27-0.45) |  |  |
| Abbreviations: RA, rheumatoid arthritis; csDMARDs: conventional synthetic disease-modifying antirheumatic drugs | | | | | | |  |
