# Supplementary material for: Association of Hospitalised Infection With Socioeconomic Status in Patients With Rheumatoid Arthritis Receiving Biologics or Tofacitinib: A Population-Based Cohort Study
Source: Front Med (Lausanne). 2021 Jul 12;8:696167. doi: 10.3389/fmed.2021.696167 (PMC8311461; doi:10.3389/fmed.2021.696167)
Supplement: Supplementary file 4 [file Table_4.DOCX]

| **Supplemental table 4. Crude and adjusted hazard ratios for the association between incident hospitalised infection and variables among 21,361 subjects with RA receiving csDMARDs** | | | | |
| --- | --- | --- | --- | --- |
|  | **HR (95% C.I.)** | **P value** | **aHR (95% C.I.)** | **P value** |
| **Age at initiating csDMARDs, years** |  |  |  |  |
| 18-45 | Ref. |  | Ref. |  |
| 45-65 | 2.07 (1.47–2.93) | <0.001 | 1.77 (1.24–2.53) | 0.002 |
| >65 | 5.66 (4.03–7.96) | <0.001 | 3.18 (2.19–4.60) | <0.001 |
| **Gender-male** | 1.87 (1.52–2.30) | <0.001 | 1.64 (1.33–2.03) | <0.001 |
| **Urbanisation status** |  |  |  |  |
| Urban | Ref. |  | Ref. |  |
| Rural | 1.28 (1.03–1.59) | 0.024 | 0.98 (0.77–1.25) | 0.894 |
| **Insured amount, New Taiwan dollars** |  |  |  |  |
| <19,200 | Ref. |  | Ref. |  |
| 19,200-22,800 | 0.94 (0.75–1.19) | 0.623 | 0.91 (0.71–1.16) | 0.430 |
| >22,800 | 0.33 (0.25–0.45) | <0.001 | 0.43 (0.31–0.60) | <0.001 |
| **Hospitalized infection within 5 years** | 4.39 (2.47–7.79) | <0.001 | 2.43 (1.35–4.36) | 0.003 |
| **Comorbidities** |  |  |  |  |
| Hypertension | 2.29 (1.87–2.80) | <0.001 | 1.34 (1.07–1.68) | 0.010 |
| Diabetes mellitus | 1.91 (1.46–2.49) | <0.001 | 1.11 (0.84–1.48) | 0.457 |
| Pulmonary disease | 2.72 (2.09–3.53) | <0.001 | 1.44 (1.09–1.91) | 0.012 |
| Chronic kidney disease | 4.45 (3.07–6.46) | <0.001 | 2.40 (1.63–3.53) | <0.001 |
| Chronic liver disease | 2.04 (1.44–2.90) | <0.001 | 1.49 (1.01–2.19) | 0.045 |
| Viral hepatitis | 2.53 (1.72–3.71) | <0.001 | 2.10 (1.38–3.21) | 0.001 |
| **Concomitant csDMARDs** |  |  |  |  |
| Prednisolone equivlent, mg/day | 1.03 (1.02–1.03) | <0.001 | 1.03 (1.03–1.03) | <0.001 |
| Methotrexate (cumulative dose/week, 2.5mg) | 1.02 (0.98–1.06) | 0.305 | 0.99 (0.95–1.02) | 0.469 |
| Sulfasalazine (cumulative dose/day, 500mg) | 1.05 (1.03–1.06) | <0.001 | 0.97 (0.95–1.001) | 0.058 |
| Leflunomide (cumulative dose/day, 50mg) | 3.75 (1.83–7.69) | <0.001 | 1.67 (0.31–8.97) | 0.550 |
| Hydroxychloroquine (cumulative dose/day, 200mg) | 1.25 (1.16–1.35) | <0.001 | 1.43 (1.27–1.60) | <0.001 |
| Cyclosporin/Azathioprin(cumulative DDD/day) | 4.46 (0.89–22.28) | 0.069 | 5.40 (0.92–31.80) | 0.062 |
| Abbreviations: RA, rheumatoid arthritis; csDMARDs: conventional synthetic disease-modifying antirheumatic drugs | | | | |
